# Development and Validation of a Prognostic Risk Model Based on Nature Killer Cells for Serous Ovarian Cancer

**DOI:** 10.3390/jpm13030403

**Published:** 2023-02-24

**Authors:** Chengxi Zhang, Chuanmei Qin, Yi Lin

**Affiliations:** 1 International Peace Maternity and Child Health Hospital, School of Medicine, Shanghai Jiao Tong University, Shanghai 200030, China; 2 Shanghai Key Laboratory of Embryo Original Diseases, Shanghai 200030, China; 3 Reproductive Medicine Center, Shanghai Sixth People’s Hospital, School of Medicine, Shanghai Jiao Tong University, Shanghai 200233, China

**Keywords:** ovarian cancer, nature killer (NK) cell, tumor microenvironment, single-cell RNA-sequencing, prognostic risk model, immunotherapy

## Abstract

Nature killer (NK) cells are increasingly considered important in tumor microenvironment, but their role in predicting the prognosis of ovarian cancer has not been revealed. This study aimed to develop a prognostic risk model for ovarian cancer based on NK cells. Firstly, differentially expressed genes (DEGs) of NK cells were found by single-cell RNA-sequencing dataset analysis. Based on six NK-cell DEGs identified by univariable, Lasso and multivariable Cox regression analyses, a prognostic risk model for serous ovarian cancer was developed in the TCGA cohort. This model was then validated in three external cohorts, and evaluated as an independent prognostic factor by multivariable Cox regression analysis together with clinical characteristics. With the investigation of the underlying mechanism, a relation between a higher risk score of this model and more immune activities in tumor microenvironment was revealed. Furthermore, a detailed inspection of infiltrated immunocytes indicated that not only quantity, but also the functional state of these immunocytes might affect prognostic risk. Additionally, the potential of this model to predict immunotherapeutic response was exhibited by evaluating the functional state of cytotoxic T lymphocytes. To conclude, this study introduced a novel prognostic risk model based on NK-cell DEGs, which might provide assistance for the personalized management of serous ovarian cancer patients.

## 1. Introduction

Ovarian cancer is a main cause of death in gynecologic cancers [1]. As the main subtype of epithelial ovarian cancer, high grade serous ovarian cancer (HGSOC) has exhibited a disappointingly low 5-year survival rate, which is only 20–30%, and has not elevated significantly in the past several decades [2]. Recurrence after traditional surgery and chemotherapy treatment is a major cause of poor clinical outcomes. As a promising novel approach, immunotherapy has been improving outcomes of various cancer types [3]. However, only a part of ovarian cancer patients have received significant benefits from immunotherapy [4,5].

As a heterogeneous system, tumor microenvironment consists of tumor cells, infiltrating immunocytes, stromal cells, and other components. The diversity of the tumor microenvironment is believed to be a crucial factor in influencing responses to immunotherapy. Increasing evidence suggests that ovarian cancer is a “immunogenic tumor” [6,7,8,9], emphasizing the importance of immunocytes in tumor microenvironment. However, in spite of immunocytes infiltration, the response rate of immune checkpoint inhibitor in ovarian cancer remain disappointingly low [10]. Thus, a deeper understanding of immunocytes in ovarian cancer is needed to help patients derive more benefits from immunotherapy. Natural killer (NK) cells are innate lymphocytes capable of killing pathogens and tumor cells nonspecifically. NK cells in tumor microenvironment is related to enhanced survival in multiple cancer types, and anti-tumor activity of NK cells can be modified by immune checkpoint blockade [11,12]. Recent research has reported the potential of NK cells as immunotherapy in a mouse model of ovarian cancer [13]. Considering the complexity and limited utilization of immunocytes like NK cells in clinical practice, a high-resolution portrait is required to achieve better clinical outcomes of ovarian cancer patients.

Heterogeneity of immunocytes in tumor microenvironment limits the effectiveness of immunotherapy. While traditional strategies are impotent to provide precise information on individual cells, single-cell RNA-sequencing (scRNA-seq) technologies can analyze immunocytes in a high-throughput and high-resolution way [14,15,16,17,18]. With the help of the scRNA-seq technologies, specific types of immunocytes in tumor microenvironment can be investigated to develop prognostic biomarkers for cancer patients. T cells [19,20] and macrophages [21,22] have recently been utilized to construct prognostic risk models for ovarian cancer, and NK cells have been utilized for other types of cancer [23,24]. However, as far as we know, there has been no research focusing on NK cells in ovarian cancer up till now. In this study, we identified differentially expressed genes (DEGs) of NK cells from a scRNA-seq dataset of serous ovarian cancer patients, and constructed a prognostic risk model based on the NK-cell DEGs. The model was then validated in external datasets. Moreover, in order to explore the possible mechanism behind this model, we performed analyses focusing on immune actives and infiltrated immunocytes in tumor microenvironment.

## 2. Materials and Methods

### 2.1. Data Collection

ScRNA-seq data from seven HGSOC samples of GSE184880 from the GEO database was used to identify NK-cell DEGs. From the UCSC Xena website (https://xenabrowser.net/, accessed on 7 October 2022), we obtained the Cancer Genome Atlas (TCGA) bulk tumor transcriptomic data and corresponding clinical information of 378 samples of serous ovarian cancer. Log_2_(FPKM + 1) was used for the TCGA transcriptomic data analysis. Based on the TCGA cohort, survival-related NK-cell DEGs were identified to construct a prognostic risk model. Afterwards, we obtained three independent microarray datasets from the GEO database for external validation of the model, namely GSE53963 (*n* = 174), GSE51088 (*n* = 117) and GSE49997 (*n* = 171). Log ratio of signal intensities was used for microarray data analysis. Only samples classified as histotype of serous ovarian cancer were used in these three GEO cohorts. Datasets described above are publicly available, and ethics approval could be obtained from original studies.

### 2.2. Analysis of scRNA-seq Dataset and Identification of NK-Cell DEGs

Firstly, an analysis of scRNA-seq dataset was performed by using R packages “Seurat” [25]. With reference to the result of Xu, J. et al. [26], we included genes that expressed no less than five single cells, cells that expressed no less than 200 genes, and excluded cells that expressed more than 40% of mitochondrial genes. ScRNA-seq data were then normalized using logarithmic normalization methods. Next, the top 1500 integrated highly variable genes were picked out, and anchors were found to integrate the data of each patient. Canonical correlation analysis (CCA) was applied for batch effect removal. After that, the principal component analysis (PCA) was performed based on the top 1500 integrated highly variable genes, so that the dimension of the scRNA-seq data could be reduced. Based on the PCA result, we picked out the first 30 significant principal components (PCs) and used them to conduct cell clustering analysis. The k-nearest neighbor (KNN) graph was constructed to determine the closest neighbors of each cell, and clusters of cells were then identified by a shared nearest neighbor (SNN) modularity optimization, and manifested by T-distributed stochastic neighbor embedding (t-SNE). Next, we identified cell types by biomarkers with reference to the result of Xu, J. et al. [26] and the PanglaoDB database [27]. DEGs of each cell type were identified by comparing with all of the other cell types using the “FindAllMarkers” function of R packages “Seurat” [25], and certain cutoff threshold values were applied, namely adjusted *p*-value < 0.01 and |log_2_(fold change)| > 0.5. Finally, NK-cell DEGs were picked out and limited to protein-coding genes, the list of which was downloaded from the HGNC database (https://www.genenames.org/, accessed on 26 December 2022).

### 2.3. Construction and Validation of the Prognostic Risk Model Based on NK-Cell DEGs

Based on the TCGA cohort, we conducted a univariate Cox regression analysis with the aim of revealing prognostic value of the NK-cell DEGs for overall survival by R package “survival” [28], and NK-cell DEGs with *p*-value < 0.05 were selected as prognostic genes. Based on these genes, we performed least absolute shrinkage and selection operator (Lasso) Cox proportional hazards regression using R package “glmnet” [29] so that overfitting could be minimized. Then we conducted 10-fold cross-validation as a means to pick out the best model, with the tuning parameter set to minimum mean cross-validated error. Thus, candidate genes with non-zero beta coefficients were identified. Lastly, on the basis of the Lasso Cox regression analysis, we performed a multivariate Cox regression analysis to evaluate theses candidate genes by the “survival” R package, and genes with *p*-value < 0.05 were identified as independent prognostic genes. Thereupon, the prognostic risk model was constructed by a linear combination of mRNA expression and the corresponding risk coefficient of these independent prognostic genes. Next, the risk score of samples in the TCGA cohort was calculated according to this model, and samples were then classified into low-risk or high-risk groups by a median value of the risk score. The Kaplan–Meier method was employed for survival analysis using R package “survminer” [30], and log-rank test was used to determine the statistical significance of the difference between low-risk and high-risk groups. To assess the predictive power of this prognostic risk model, area under the curve (AUC) was calculated using R package “survivalROC” [31]. Finally, three independent cohorts were obtained from the GEO database to validate this model’s ability of predicting prognosis.

### 2.4. Functional Enrichment Analysis

Gene Ontology (GO) and Kyoto Encyclopedia of Genes and Genomes (KEGG) are two widely used databases for functional enrichment analysis. In this study, we conducted GO and KEGG functional enrichment analysis with the help of R package “clusterProfiler” [32]. GO analysis was annotated based on R package “org.Hs.eg.db” [33]. The latest online KEGG database was accessed for KEGG analysis. Adjusted *p*-value < 0.05 was considered significant.

### 2.5. Immune Activities Assessment

Seven clusters of metagenes (STAT1, HCK, IgG, LCK, MHC-I, MHC-II, and Interferon) have been widely utilized to evaluate immune and inflammatory activities in tumor microenvironment [34]. We used gene set variation analysis (GSVA) to explore expression of these metagenes in the TCGA cohort by R package “GSVA” [35], and then conducted correlation analysis for the GSVA scores and the risk score. *p*-value < 0.05 was considered significant. Next, we conducted estimation of stromal and immune cells in malignant tumor tissues using expression data (ESTIMATE) algorithm with the purpose of assessing the infiltration level of immunocytes by R package “estimate” [36]. Immune score, stromal score, and ESTIMATE score of the ESTIMATE algorithm were calculated in the TCGA cohort, and in order to compare the difference between low-risk and high-risk groups, we conducted *t*-test or Mann–Whitney *U* test.

### 2.6. Quantity and Functional State of Immunocytes Evaluation

Cell-type identification by estimating relative subsets Of RNA transcripts (CIBERSORT) is a useful method for obtaining the characteristics of cell types based on gene expression profiles [37]. We conducted the CIBERSORT algorithm in the TCGA cohort to asses infiltration levels of 22 types of immunocytes. Afterwards, we applied tumor immune dysfunction and exclusion (TIDE). Fu et al. introduced this novel algorithm, which is able to predict the response to immune checkpoint blocking therapy by analyzing the dysfunction and exclusion profiles of cytotoxic T cells [38,39]. T cell dysfunction score, T cell exclusion score, and TIDE score of the TIDE algorithm were calculated using python package “TIDEpy” [38,39]. *t*-test or Mann–Whitney *U* test was performed to compare different results between low-risk and high-risk groups.

### 2.7. Statistical Analyses

Categorized variables between low-risk and high-risk groups were compared by *t*-test or Mann–Whitney *U* test. The *p*-value < 0.05 was set as a significant threshold. The Benjamini–Hochberg (BH) method was applied to adjust the *p*-value for multiple testing. Linear correlation between two sets of numerical variables was measured by the Pearson correlation method, which calculates a value between −1 and 1 to quantify correlation. In the process of data analyses and figure production, we mainly used R software version 4.2.1 (http://www.R-project.org, accessed on 27 September 2022).

## 3. Results

### 3.1. Identification of NK-Cell DEGs by scRNA-seq Dataset Analysis

**Figure 1:**
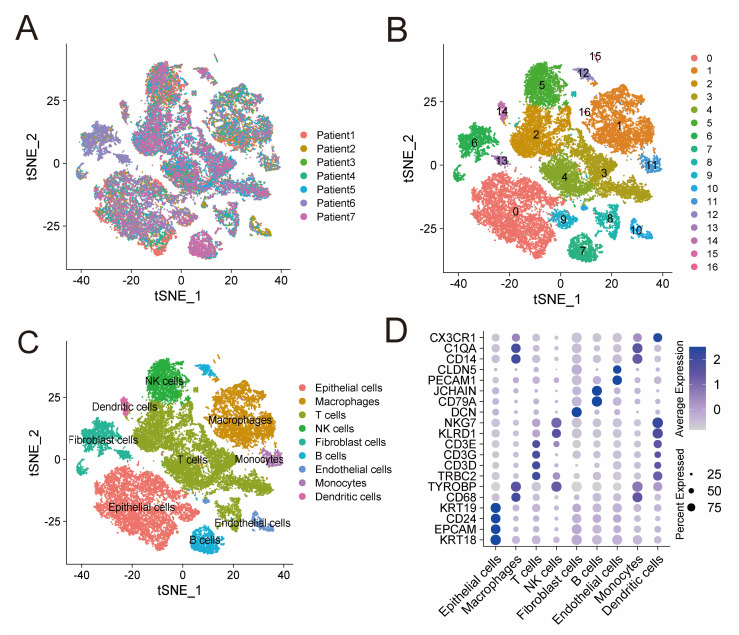
ScRNA-seq analysis identifies NK-cell DEGs. (**A**) t-SNE plot of 33,546 cells from seven treatment-naive HGSOC patients. (**B**) Seventeen clusters identified in the 33,546 cells. (**C**) Cell types annotated by marker genes. (**D**) Bubble plot shows the expression level of marker genes in different cell types. Color intensity of the bubble represents average expression level and size of the bubble represents percentage of cells expressing the gene in each cell type. ScRNA-seq: single-cell RNA-sequencing; DEGs: differentially expressed genes; and HGSOC: high grade serous ovarian cancer.

ScRNA-seq data of GSE184880 provided us with gene expression profiles of 33,546 cells from seven treatment-naive HGSOC patients (Figure 1A). Based on that, we conducted PCA using the top 1500 variable genes, and cells were thus classified into 17 clusters (Figure 1B). These clusters were then annotated with reference to established marker genes. Figure 1C demonstrates identified cell types, including NK cells (marked by KLRD1 and NKG7), T cells (marked by TRBC2, CD3D, CD3G and CD3E), monocytes (marked by CD14 and C1QA), epithelial cells (marked by EPCAM, CD24, KRT18 and KRT19), B cells (marked by CD79A and JCHAIN), macrophages (marked by CD68 and TYROBP), endothelial cell types (marked by PECAM1 and CLDN5), dendritic cells (marked by CX3CR1), and fibroblast cells (marked by DCN). The expression level of these marker genes in different cell types are shown in Figure 1D. Cells in cluster 5 were annotated as NK cells, subsequently, 576 protein-coding genes were identified as NK-cell DEGs (Table S1). Functional enrichment analysis, including GO and KEGG, indicated that these 576 genes are mainly enriched in immune features, such as leukocyte mediated immunity, cytokine receptor binding, and natural killer cell mediated cytotoxicity (Figure S1).

### 3.2. Development of a Prognostic Risk Model Based on the NK-Cell DEGs

**Figure 2:**
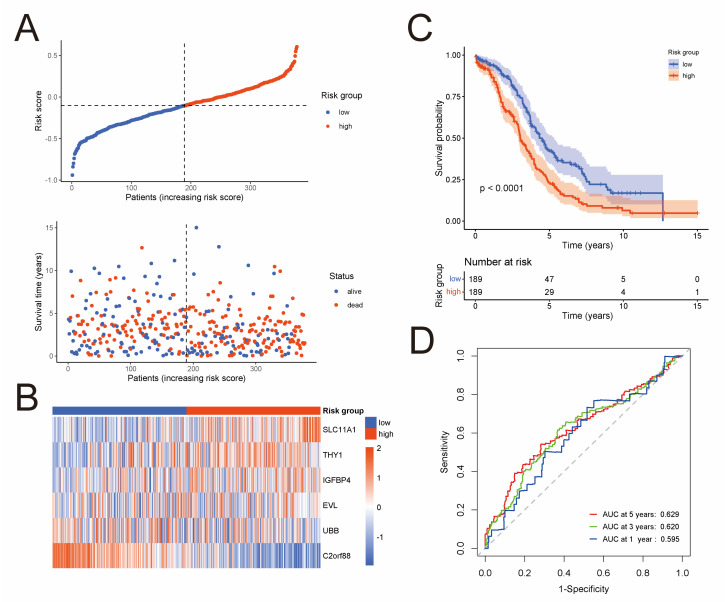
Development of a prognostic risk model based on the NK-cell DEGs in the TCGA cohort (*n* = 378). (**A**) Distribution of risk scores and survival status. (**B**) Heatmap shows expression level of the six genes forming the prognostic risk model. (**C**) Kaplan–Meier curves of survival analysis compares overall survival between low-risk and high-risk groups. (**D**) ROC curves of the prognostic risk model for predicting risk of death at 1, 3, and 5 years.

To develop a prognostic risk model based on the 576 NK-cell DEGs, we firstly perform a univariate Cox regression analysis in the TCGA cohort, and 57 NK-cell DEGs were found to be significantly related to overall survival (Table S2). Next, Lasso Cox regression analysis was conducted as described in the Materials and Methods on these 57 prognostic NK-cell DEGs (Figure S2A,B), and 19 genes were picked out, namely CD38, SLC11A1, SLAMF7, GBP1, FOSB, JCHAIN, IGFBP4, THY1, CXCL13, IFI27, CXCL10, UBB, BTN3A2, CMC1, C2orf88, CLDN4, EVL, GZMM, and TMSB4X. Lastly, we conducted a multivariate Cox regression analysis (Figure S2C), and six most prognostic NK-cell DEGs were identified to form the prognostic risk model: risk score = (0.236 × SLC11A1 expression) + (0.048 × THY1 expression) + (0.017 × IGFBP4 expression) + (−0.016 × EVL expression) + (−0.045 × UBB expression) + (−0.126 × C2orf88 expression). The risk score of each sample in the TCGA cohort was calculated according to this model, and the median risk score was used to classify samples into low-risk (*n* = 189) or high-risk (*n* = 189) groups. Figure 2A exhibits the distribution of risk scores and survival status of the TCGA cohort. Heatmap demonstrates expression level of these six genes forming the prognostic risk model (Figure 2B). Then, a Kaplan–Meier analysis was conducted and the result revealed that the low-risk group had significantly superior overall survival than the high-risk group (Figure 2C). To assess the predictive accuracy of this model, area under the ROC curves for overall survival was calculated, and 1-, 3-, and 5-year mean AUC values are 0.595, 0.620, and 0.629, respectively (Figure 2D).

### 3.3. External Validation of the Prognostic Risk Model in Independent Cohorts

**Figure 3:**
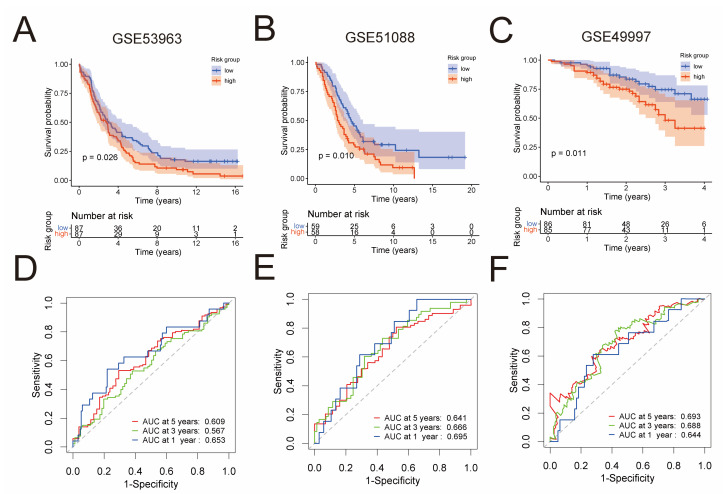
External validation of the prognostic risk model in three independent GEO cohorts. Kaplan–Meier analysis compared overall survival of the GSE53963 (*n* = 174) (**A**), GSE51088 (*n* = 117) (**B**), and GSE49997 (*n* = 171) (**C**) cohort between low-risk and high-risk groups. ROC curves of the prognostic risk model for predicting risk of death at 1, 3, and 5 years in the GSE53963 (**D**), GSE51088 (**E**), and GSE49997 (**F**) cohort.

In order to validate the predictive capability of the prognostic risk model, three independent GEO cohorts were used, namely GSE53963 (*n* = 174), GSE51088 (*n* = 117), and GSE49997 (*n* = 171). By the Kaplan–Meier analysis, we found that the low-risk group had significantly superior overall survival than the high-risk group in each of these cohorts (Figure 3A–C). To assess the predictive accuracy of the prognostic risk model, area under the ROC curves for overall survival was calculated. In the GSE53963 cohort, mean AUC values of 1, 3, and 5 years are 0.653, 0.567, and 0.609, respectively (Figure 3D). In the GSE51088 cohort, mean AUC values of 1, 3, and 5 years are 0.695, 0.666, and 0.641, respectively (Figure 3E). Moreover, in the GSE49997 cohort, mean AUC values of 1, 3, and 5 years are 0.644, 0.688, and 0.693, respectively (Figure 3F).

### 3.4. The Prognostic Risk Model Acted as an Independent Prognostic Factor

In order to rule out the confounding effect, we analyzed six clinical characteristics together with the prognostic risk model in the TCGA cohort, namely age, tumor status, clinical stage, venous invasion, lymphatic invasion, and residual disease. Firstly, we performed a univariable Cox regression analysis on these characteristics to identify whether they were related to overall survival of serous ovarian cancer patients. Results showed that four characteristics were significantly associated with overall survival, namely age, tumor status, residual disease, and risk group. Subsequently, we conducted a multivariable Cox regression analysis on these four characteristics, and the result demonstrated tumor status and risk group as independent prognostic factors (Table 1). The characteristic tumor status described the state of a patient’s neoplasm in the TCGA cohort by using the value “Tumor free” or “With tumor”. To summarize, these results validated the prognostic risk model as an independent factor of predicting prognostic risk in serous ovarian cancer.

### 3.5. Functional Enrichment Analysis of Genes Correlated with the Prognostic Risk Model

**Figure 4:**
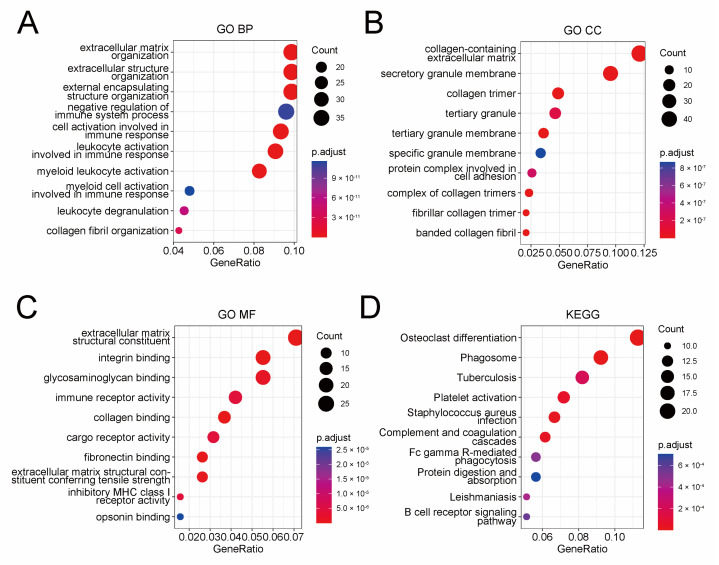
Functional enrichment analysis of 398 genes closely correlated with the prognostic risk model identified by Pearson correlation analysis. Dot plot of GO BP (**A**), GO CC (**B**), GO MF (**C**), and KEGG (**D**) analysis of these 398 genes. GO BP: biological process of gene ontology; GO CC: cellular components of gene ontology; GO MF: molecular function of gene ontology; KEGG: Kyoto encyclopedia of genes and genomes.

To elucidate the underlying mechanism of the prognostic risk model, we conducted a functional enrichment analysis of genes, which were found to be correlated with the model. At first, a correlation analysis was performed in the TCGA cohort to pick out protein-coding genes closely correlated with the risk score (Pearson |R| > 0.3, *p*-value < 0.01), and a total of 398 genes were selected (Table S3). Then, we performed GO and KEGG enrichment analysis for these genes (Figure 4A–D). The GO analysis indicated that these genes were mostly related to extracellular structure organization, while the KEGG analysis indicated involvement in phagosome pathway. Besides that, immune features showed significance in both GO and KEGG analysis, such as negative regulation of immune system process, immune receptor activity, and tuberculosis. Collectively, these results suggested nonnegligible association between the prognostic risk model and the tumor microenvironment of serous ovarian cancer, especially the immune microenvironment.

### 3.6. Assessment of Immune Activities in Tumor Microenvironment

**Figure 5:**
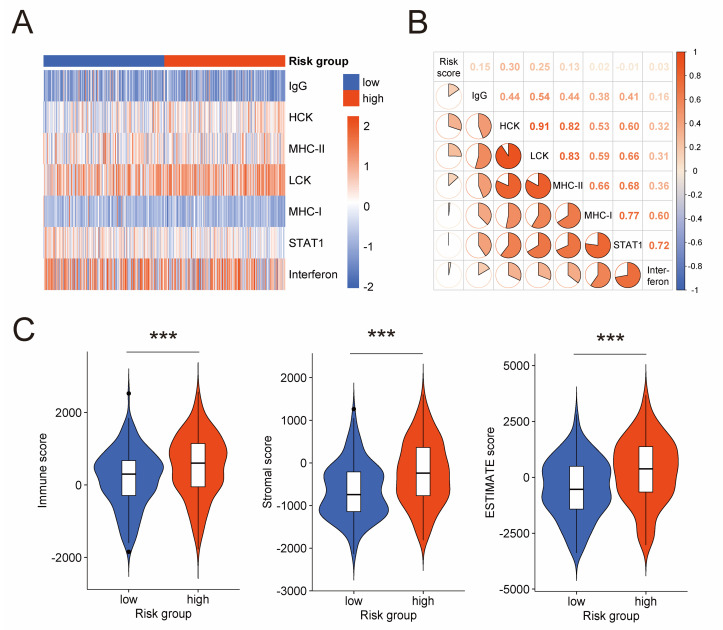
Association between the prognostic risk model and immune activities in tumor microenvironment. (**A**) Heatmap shows the GSVA score of seven clusters of metagenes representing different immune and inflammatory activities. (**B**) Correlogram exhibits the correlation between the risk score and GSVA scores of these metagenes. (**C**) Difference of immune score, stromal score, and ESTIMATE score between low-risk and high-risk groups by ESTIMATE algorithm. GSVA: gene set variation analysis; ESTIMATE: estimation of stromal and immune cells in malignant tumor tissues using expression data. ***: *p*-value < 0.001.

With a focus on the association between the prognostic risk model and tumor immune microenvironment, we performed analyses on the activities of immune and inflammation, and infiltration of immunocytes. Firstly, we explored the relation between the risk score and seven clusters of metagenes (IgG, MHC-I, MHC-II, HCK, Interferon, LCK, and STAT1). These metagenes introduced by Rody, A. et al. have been extensively used to represent various immune and inflammatory activities [34]. GSVA was conducted to analyze expression of these metagenes in the TCGA cohort (Figure 5A). Then, correlation analysis for the risk score and GSVA scores was carried out (Figure 5B), which disclosed positive correlation between the risk score and four clusters of metagenes, namely IgG (Pearson |R| = 0.15, *p*-value = 0.003), HCK (Pearson |R| = 0.30, *p*-value < 0.001), LCK (Pearson |R| = 0.25, *p*-value < 0.001), and MHC-II (Pearson |R| = 0.13, *p*-value = 0.010). These positive correlations further validated the association between the prognostic risk model and the tumor immune microenvironment of serous ovarian cancer. Next, we conducted ESTIMATE algorithm, which uses gene expression signatures to infer fraction of immunocytes in tumor samples. We found that the high-risk group had a higher immune score than the low-risk group (Figure 5C), suggesting more immunocytes infiltrating in the high-risk group. This result is consistent with the immunogenicity characteristic of ovarian cancer [6,7,8,9] and impelled us to dig deeper into these immunocytes.

### 3.7. Association of the Prognostic Risk Model with Quantity and Functional State of Immunocytes

**Figure 6:**
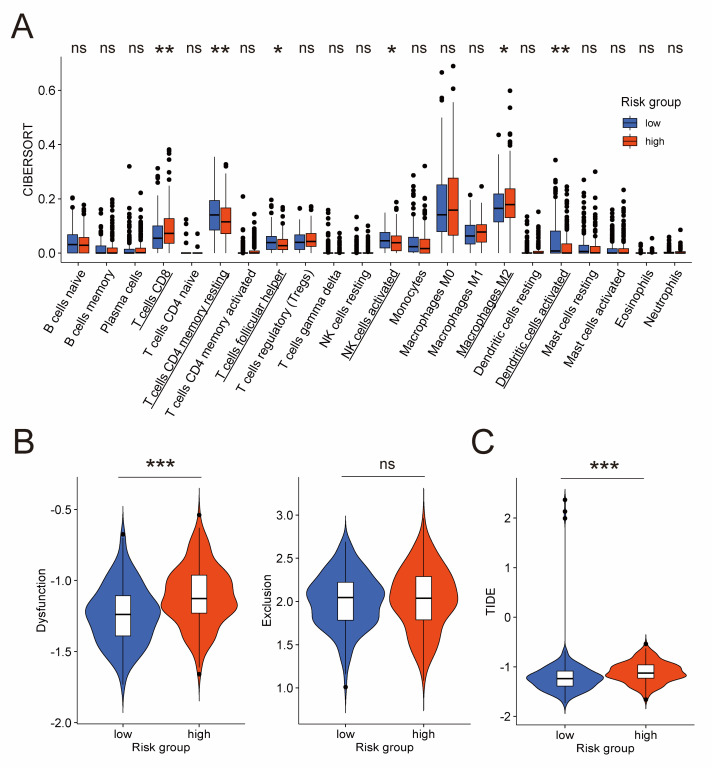
Evaluation of quantity and functional state of immunocytes in the TCGA cohort. (**A**) Difference of infiltration level of 22 immunocyte types between low-risk and high-risk groups by CIBERSORT algorithm. Names of cell types with significant differences are underlined. Difference of T cell dysfunction and exclusion score (**B**), and TIDE score (**C**) between low-risk and high-risk groups by TIDE algorithm. CIBERSORT: cell-type identification by estimating relative subsets of RNA transcripts; TIDE: tumor immune dysfunction and exclusion. *: *p*-value < 0.5; **: *p*-value < 0.01; ***: *p*-value < 0.001; ns: not significant.

Considering the importance of immunocytes like NK cells in tumor [11,12,13], we performed a detailed investigation into specific types of immunocytes. At first, we applied CIBERSORT algorithm in the TCGA cohort to calculate infiltration levels of 22 types of immunocytes in the tumor microenvironment. The result exhibited that, compared to the low-risk group, the high-risk group had lower fraction of activated NK cells, resting CD4^+^ memory T cells, follicular helper T cells, and activated dendritic cells, but higher fraction of CD8^+^ T cells and M2 macrophages (Figure 6A). NK cells and CD8^+^ T cells (often called cytotoxic T lymphocytes) are both generally considered as anti-tumor immunocytes [40,41,42,43], but the CIBERSORT algorithm revealed a complicated result with less NK cells and more CD8^+^ T cells in the high-risk group. A possible explanation was provided by TIDE, an algorithm for evaluating the dysfunction and exclusion profiles of cytotoxic T lymphocytes. As is shown in Figure 6B, the T cell dysfunction score of the high-risk group was significantly higher, whereas the T cell exclusion score exhibited no significant difference. This result indicated more dysfunction of cytotoxic T lymphocytes in the high-risk group. However, as for NK cells, they have also been reported as dysfunctional in ovarian cancer [44,45]. A widely used algorithm for evaluating the functional sate of NK cells is currently unavailable as far as we know. Thus, further research is required in the future to figure out whether less NK cells in the high-risk group, which was demonstrated by the CIBERSORT algorithm, is associated with worse prognosis.

Besides that, the TIDE score of the TIDE algorithm, which takes both T cell dysfunction and exclusion into consideration to predict immunotherapeutic response, is significantly higher in the high-risk group compared to the low-risk group (Figure 6C). This result suggested that the high-risk group was less likely to benefit from immunotherapy owing to more dysfunction of cytotoxic T lymphocytes in the tumor microenvironment, which is consistent with the view that the immunotherapeutic response of ovarian cancer patients is disappointingly poor [4,5].

## 4. Discussion

Recently, the role of tumor microenvironment in progression and treatment of ovarian cancer is increasingly appreciated. NK cells, a portion of the tumor microenvironment, have been developed as a promising biomarker for prognosis prediction in cutaneous melanoma [23] and lung adenocarcinoma [24]. Cursons et al. investigated marker genes of NK cells and revealed their association with the prognosis of cutaneous melanoma [23]. Song et al. introduced a signature based on NK cell marker genes and discovered that lung adenocarcinoma patients with lower risk scores had superior prognosis, and could benefit more from immune checkpoint blockade therapy [24]. Inspired by their promising findings, this study tried to explore the value of NK cells in predicting prognostic risk in serous ovarian cancer, which has not been reported previously as far as we know. With a combination of single-cell and bulk RNA-sequencing datasets, we developed a prognostic risk model from NK-cell DEGs. This model was then validated in independent cohorts. An underlying mechanism was explored, and we found that not only quantity, but also functional state of immunocytes in tumor microenvironment could affect prognostic risk. In addition to superior prognosis, we revealed that a lower risk score of this model was also associated with better response to immunotherapy.

By scRNA-seq technology, we constructed the prognostic risk model based on six NK-cell DEGs, namely SLC11A1, THY1, IGFBP4, EVL, UBB, and C2orf88. Most of these genes have been reported to be associated with ovarian cancer or NK cells. Hedges et al. demonstrated the expression of SLC11A1 in NK cells, which could enhance NK-cell activation [46]. Connor et al. suggested that THY1 (also known as CD90) was a marker of cancer stem cells and could promote proliferation and self-renewal ability of ovarian cancer cells [47]. However, Chen et al. got a contrary result, indicating that THY1 was a tumor suppressor gene by inhibiting stemness properties of ovarian cancer cells [48]. IGFBP4 was significantly elevated in all stages of ovarian cancer patients, even in early stage without elevated CA125 [49]. Moreover, elevated expression of IGFBP4 was associated with worse overall survival in ovarian cancer patients who received platin chemotherapeutic regimen [50]. EVL was reported to mediate adhesion and cytotoxicity of NK cells [51]. Inhibited UBB expression was associated with poorer survival outcomes in ovarian cancer [52,53]. C2orf88 was related to increased number of filopodia in HeLa cells, which could promote cancer progression [54]. However, as far as we know, the relation between C2orf88 and ovarian cancer or NK cells has not been studied. Nevertheless, these reports suggest that further research on these six genes might provide molecular mechanisms of NK-cell activities in serous ovarian cancer.

The prognostic model developed from the TCGA cohort was then validated in three independent GEO cohorts and evaluated together with clinical characteristics to rule out the confounding effect. These favorable results inspired us to explore the underlying mechanism. The GO and KEGG analysis was performed on genes closely correlated to this model, and the result suggested that tumor microenvironment, especially the immune microenvironment, might play a vital role in serous ovarian cancer. Focusing on tumor immune microenvironment, we firstly investigated the association between this model and activities of immune and inflammation. Four clusters of metagenes (IgG, MHC-II, HCK and LCK) representing various immune and inflammatory activities were identified to be positively correlated with this model. IgG is a product of B cells [34,55,56]. MHC-II expression is restricted to professional antigen-presenting cells [34,57]. HCK cluster includes genes like hemopoietic cell kinase (HCK), CD163, and CCR1, which are mainly expressed in the monocyte/myeloid lineages and B lymphocyte lineages [34,58]. LCK cluster contains genes like lymphocyte-specific kinase (LCK), T-cell receptor α, and T-cell receptor β, and these genes play vital roles in T cells [34,59,60]. Collectively, these positive correlations revealed active immune and inflammatory activities in tumor microenvironment of serous ovarian cancer. Furthermore, we compared the abundance of immunocyte infiltration between low-risk and high-risk groups by ESTIMATE algorithm. The immune score of the ESTIMATE algorithm in the high-risk group suggested a higher infiltration level of immunocytes than the low-risk group. To summarize, these results were consistent with findings regarding ovarian cancer as a “immunogenic tumor” [6,7,8,9], and pointed out the need to dig deeper into tumor immune microenvironment.

In spite of active immune activities, the survival rate of ovarian cancer patients remain disappointingly low [2]. Therefore, we performed a detailed inspection into immunocytes in tumor microenvironment. CIBERSORT algorithm was used to analyze fraction of 22 types of immunocytes, and the low-risk group exhibited a higher fraction of activated NK cells, but a lower fraction of CD8^+^ T cells than the high-risk group. Garzetti et al. reported that the quantity of NK cells in peripheral blood was significantly lower in ovarian cancer patients during disease progression [41]. Hoogstad-van Evert et al. obtained a similar result, demonstrating an association between more NK cells in the ascites and an increased survival of ovarian cancer patients [61]. As for CD8^+^ T cells, they are generally considered as anti-tumor effectors [42,43], but a contrary result was also reported, whereby more CD8^+^ T cells were related to an advanced stage of ovarian cancer [62]. These confusing findings indicated that, besides quantity, other characteristics of immunocytes were required to be dissected. Dysfunction of immunocytes has been observed in several tumors, and could lead to both pro-tumor and anti-tumor activities [63,64]. Focusing on the functional state of immunocytes, we conducted TIDE, an algorithm for analyzing T cell dysfunction and exclusion. The result revealed a higher T cell dysfunction score in the high-risk group, suggesting that cytotoxic T lymphocytes were in dysfunctional state. However, as for NK cells, their dysfunction in ovarian cancer was also reported [44,45]. Therefore, it remains uncertain whether more NK cells in the low-risk group demonstrated by the CIBERSORT algorithm is related to superior prognosis. As far as we know, extensively used algorithm for evaluating the functional state of NK cells is currently unavailable, so more efforts are needed in the future.

In addition, the TIDE algorithm demonstrated the association between the prognostic risk model and immunotherapeutic response. As is described above, the TIDE score of the TIDE algorithm was significantly higher in the high-risk group compared to the low-risk group, which indicated less potential for the high-risk group to benefit from immunotherapy. This is consistent with the view that ovarian cancer patients respond poorly to immunotherapy [4,5], and may be ascribed to more dysfunction of cytotoxic T lymphocytes in the high-risk group according to the TIDE algorithm [38,39]. In recent years, NK cells are increasingly considered important for immunotherapy [11,65], impelling us to explore their role in predicting the immunotherapeutic response of ovarian cancer patients. Interactions between T cells and NK cells were widely reported [66,67,68,69], and two genes forming the prognostic risk model were found to participate in the regulating function of both T cells and NK cells, namely SLC11A1 [46,70,71,72] and EVL [51,73,74,75]. Thus, we infer that immunotherapeutic response might also be predicted by evaluating the functional state of NK cells. However, no meaningful results have been achieved by us yet, and future research is required to investigate this speculation.

In conclusion, based on NK-cell DEGs identified by scRNA-seq analysis, a prognostic risk model was developed for serous ovarian cancer in this study, which also exhibited the potential for predicting immunotherapeutic response. However, this study has several limitations. Firstly, the NK-cell specificity of the prognostic risk model is limited because the model was developed from DEGs, which are not necessarily specific marker genes of NK cells. Secondly, the value of this model is limited due to the complexity of tumor microenvironment. Besides, the model should be validated in larger cohorts with more detailed clinical information. Lastly, the underlying mechanism of this model requires to be investigated in basic experiments. Collectively, the prognostic risk model might provide assistance for predictions of prognostic risk and immunotherapeutic response so as to improve personalized management of serous ovarian cancer patients, and further research is required to exploit more clinical value from immunocytes like NK cells in tumor microenvironment.

## Figures and Tables

**Table 1:** Univariable and multivariable Cox regression analyses of clinical characteristics together with prognostic risk model in the TCGA cohort.

Characteristics	Univariable Analysis		Multivariable Analysis	
	HR (95% CI)	*p*-Value	HR (95% CI)	*p*-Value
Age				
≤60	reference		reference	
>60	1.36 (1.05–1.76)	*	1.20 (0.89–1.60)	ns
Tumor status				
Tumor free	reference		reference	
With tumor	8.39 (4.55–15.46)	***	9.18 (4.49–18.79)	***
Clinical stage				
I + II	reference			
III + IV	2.13 (0.95–4.81)	ns		
Venous invasion				
No	reference			
Yes	0.90 (0.49–1.65)	ns		
Lymphatic invasion				
No	reference			
Yes	1.39 (0.82–2.34)	ns		
Residual disease				
≤10 mm	reference		reference	
>10 mm	1.50 (1.12–2.00)	**	1.19 (0.87–1.63)	ns
Risk group				
Low	reference		reference	
High	1.80 (1.39–2.34)	***	1.61 (1.20–2.15)	**

*: *p*-value < 0.5; **: *p*-value < 0.01; ***: *p*-value < 0.001; ns: not significant.

## Data Availability

Data used in this work is available in the TCGA database (https://www.cancer.gov/tcga, accessed on 7 October 2022) and the GEO database (https://www.ncbi.nlm.nih.gov/geo/, accessed on 29 December 2022) under accession numbers GSE184880, GSE53963, GSE51088 and GSE49997.

## Supplementary Materials

The following supporting information can be downloaded at: https://www.mdpi.com/article/10.3390/jpm13030403/s1, Figure S1. Functional enrichment analysis of the 576 NK-cell DEGs. Dot plot of GO BP (A), GO CC (B), GO MF (C), and KEGG (D) analysis. Color of bubbles represents adjusted *p*-value and size of bubbles represents the count of genes in each signaling pathway; Figure S2. Construction of the prognostic risk model in the TCGA cohort. (A) Coefficient profiles of the 19 NK-cell DEGs identified by Lasso regression analysis. (B) Ten-fold cross-validation for selecting parameter in the Lasso regression analysis. (C) Multivariable Cox regression analysis of the six genes forming the model. *: *p*-value < 0.5; **: *p*-value < 0.01; ***: *p*-value < 0.001; ns: not significant; Table S1. The 576 NK-cell DEGs identified from the scRNA-seq dataset GSE184880; Table S2. The 57 NK-cell DEGs associated with overall survival identified by univariable Cox regression analysis in the TCGA cohort; Table S3. The 398 genes that closely correlated with the prognostic risk model identified by Pearson correlation analysis.
