# Supplementary figures and images for: Development and Validation of a Prognostic Risk Model Based on Nature Killer Cells for Serous Ovarian Cancer
Source: J Pers Med. 2023 Feb 24;13(3):403. doi: 10.3390/jpm13030403 (PMC10055736; doi:10.3390/jpm13030403)

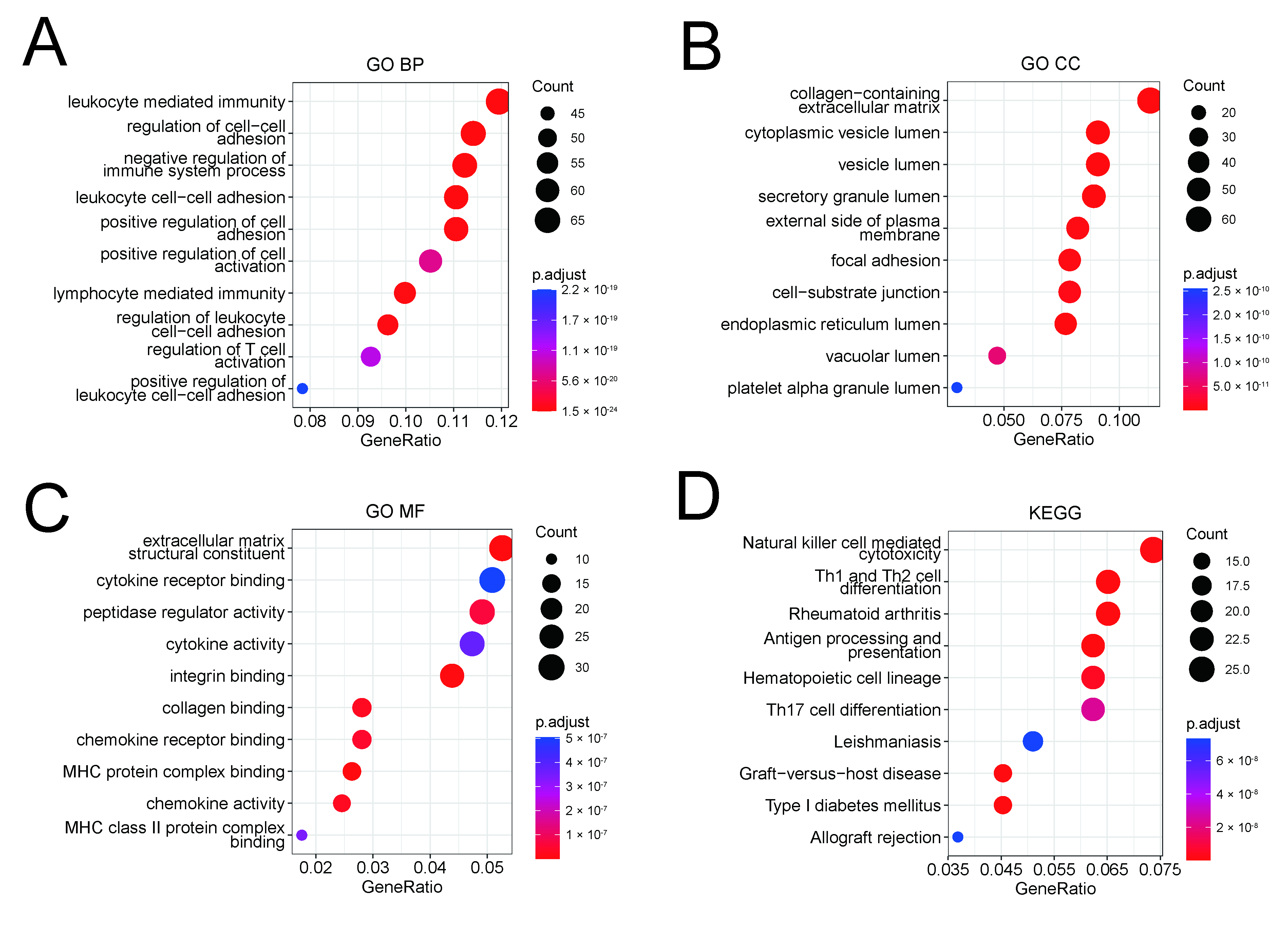

Supplement: Supplementary file 1 [file jpm-13-00403-s001.zip › Figure S1.tif]

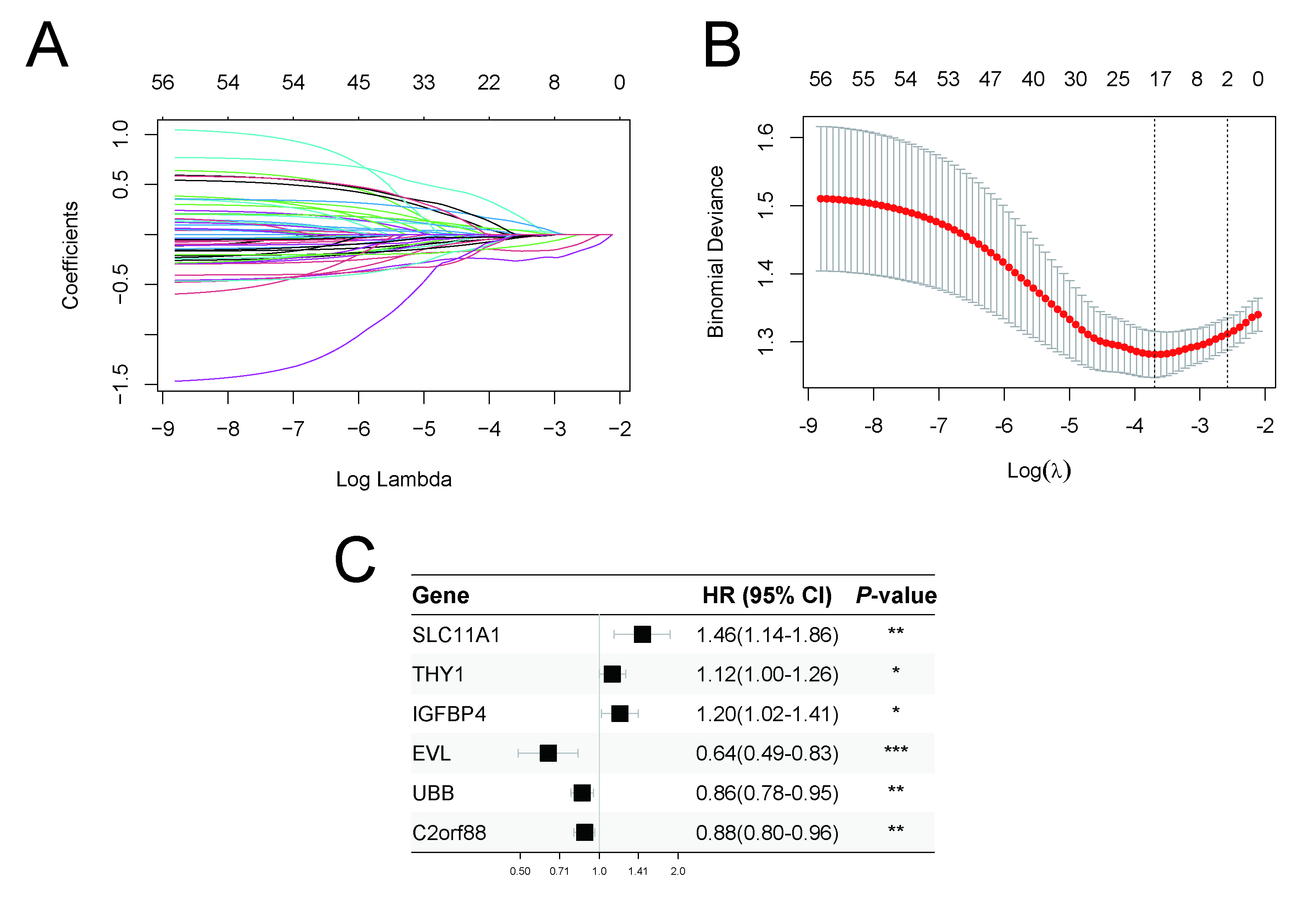

Supplement: Supplementary file 1 [file jpm-13-00403-s001.zip › Figure S2.tif]
